# Optimisation of Volume Flow Rates when Using Endovascular Shunting Techniques: An Experimental Study in Different Bench Flow Circuits

**DOI:** 10.1016/j.ejvsvf.2022.11.002

**Published:** 2022-11-19

**Authors:** Johan Millinger, Daniel Bengtsson, Marcus Langenskiöld, Andreas Nygren, Klas Österberg, Joakim Nordanstig

**Affiliations:** a Department of Molecular and Clinical Medicine, Institute of Medicine, University of Gothenburg, Gothenburg, Sweden; b Department of Vascular Surgery, Sahlgrenska University Hospital, Gothenburg, Sweden; c Department of Cardiothoracic Surgery, Sahlgrenska University Hospital, Göteborg, Sweden; d Institute of Clinical Sciences, Sahlgrenska Academy, University of Gothenburg, Gothenburg, Sweden; e Department of Anaesthesiology and Intensive Care Medicine, Sahlgrenska University Hospital, Gothenburg, Sweden

**Keywords:** Aorta/surgery, Endovascular procedures, Ischaemia, Lower extremity/blood supply, Perfusion/methods, Vascular surgical procedures

## Abstract

**Objective:**

Acute tissue ischaemia may arise due to arterial emergencies or during more complex vascular procedures and may be mitigated by temporary shunting techniques. Endovascular shunting (ES) techniques enable percutaneous access and shunting from the donor artery without the need to completely interrupt the arterial flow in the donor artery. An endoshunt system may also cover longer distances than most conventional shunts. The aim was to investigate and optimise the flow rates in different endovascular shunt systems.

**Methods:**

Step 1: The flow capacity of different ES configurations was compared with the flow capacity of a 9 Fr Pruitt–Inahara shunt (PIS). An intravenous bag with 0.9% NaCl, pressurised to 90 mmHg, was connected simultaneously to a PIS and to one of the tested ES configurations. The two shunt systems were then opened at the same time. The delivered fluid volumes from the shunt systems were collected and measured. The volume flow rate was subsequently calculated. Steps 2 and 3: Within a heart lung machine circuit, pressure–flow charts were constructed for the individual ES components and for the fully connected optimised endoshunt systems. The flow rate was increased in steps of 40–50 mL/min while monitoring the driving pressure, enabling the creation and comparison of the pressure–flow charts for the individually tested components. In total, seven individual inflow and outflow potential ES components were investigated with inflow and outflow diameters ranging from 6 to 15 Fr.

**Results:**

ES systems based on standard donor introducers led to substantially lower volume flow than the corresponding PIS volume flow, whereas ES systems based on dedicated 6 or 8 Fr dialysis access introducers (Prelude Short Sheet, Merit Medical) matched PIS flow rates. The introduction of 30 cm long ¼′′ perfusion tubing within the ES system did not affect volume flow for any of the tested ES configurations.

**Conclusion:**

Endoshunting techniques can match PIS volume flow rates over short and long distances. The achieved ES flow rate is highly dependent on the components used within the ES system.

## Highlights

• Endovascular shunting techniques have emerged as versatile approaches to manage ischaemia during vascular emergencies and procedures but remain poorly studied.
• In this study the flow rates in different endovascular shunt systems were investigated. Flow capacity differences were noted both between individual devices and different endoshunt systems.
• Only optimised endoshunt circuits matched or outperformed the corresponding flow capacity of a commercially available conventional vascular shunt.
• These observations may guide vascular surgeons to select more optimised endoshunting approaches that ultimately may limit end organ damage in many clinical scenarios.

## Introduction

Acute tissue ischaemia and subsequent reperfusion can cause permanent tissue damage, compartment syndrome and transient or permanent loss of organ function. In addition, reperfusion may cause systemic injuries to the heart, lung and kidneys.^1^^,^^2^ The most common causes of ischaemia are trauma and different arterial emergencies, but tissue ischaemia may also occur during more complex elective and emergency endovascular or open arterial procedures.

A common technique to deal with this problem within vascular surgery is by using temporary shunting of blood via various types of plastic tube systems.^3^^,^^4^ The first attempts to solve the problem of ischaemia caused by vascular trauma by using vascular shunts were described at the beginning of the 20th century. Although anecdotally described during both the first and the second world wars, it was not until the 1950–1960s that vascular shunt techniques gained popularity and eventually became a tool used on a more regular basis.^5^ All conventional shunting techniques however require open exposure of a suitable donor artery, and also normally lead to a complete interruption of blood flow to the tissue or body region that is being supplied by the donor artery.^4^ This greatly limits the possible arteries that can be used as donor arteries during shunting.

Endovascular shunting (ES) techniques may overcome these problems. The technique enables shunting of blood from a percutaneously accessed donor artery, and may cover large distances between donor and target artery; it commonly also eliminates the need to completely interrupt blood flow in the donor artery. This in turn minimises the need for additional surgical trauma, increases the overall flexibility of shunting techniques, and increases the number or suitable donor arteries, whenever shunting is a preferred step to reduce end organ damage during vascular interventions. In the clinic, the brachial artery has been used as a donor when shunting extensive lower extremity vascular injuries and to temporarily perfuse renal arteries during more complex open aortic surgery. The ES technique was first scientifically described by Österberg et al. in 2014.^6^ In their article, the experimental flow capacity of some ES shunt systems was also studied and compared with the corresponding flow rates observed for the Pruitt–Inahara shunt (PIS), which remains one of the most widely used and studied conventional shunts in vascular surgery.^7^, ^8^, ^9^ Using a quite simple experimental set up, this study indicated that the flow rate of the ES system was not able to completely match that of the PIS, which in turn represents a potentially important drawback with ES techniques.^6^ Also, compared with conventional shunting techniques, the efficacy and safety of ES techniques has not yet been extensively studied.

The aim of this exploratory study was to investigate and optimise the volume flow rates in different endovascular shunt systems, with the main hypothesis that an optimised endoshunt set up would be able to match − or even outperform − the corresponding flow capacity of conventional shunts commonly used in vascular surgery.

## Materials and methods

### Overall study design

This experimental bench test study was performed in three steps. In the first step, a range of tentatively suitable short and long range endoshunt system volume flow capacities were studied and compared with the corresponding flow volumes of the 9 Fr Pruitt–Inahara carotid shunt within a simple bench test set up using a pressurised intravenous bag with saline solution at a fixed pressure of 90 mmHg. In the second step, the different integral components of the more promising endoshunt systems were investigated in an experimental cardiopulmonary bypass circuit that allowed the mean pressure within the flow circuit to be controlled and gradually modified (i.e., mimicking different levels and variations in mean arterial pressure). The tested components during this second step were selected mainly based on the observed results in step 1, but also included *a priori* were a few additional shunt component candidates that were suggested by the perfusionist collaborator (DLP 8 Fr One Piece Paediatric Arterial Cannula, Medtronic, DLP 10 Fr One Piece Paediatric Arterial Cannula, Medtronic, DLP 15 Fr Coronary Ostial Perfusion Cannula, Medtronic and the 8 Fr Super Arrow-Flex sheath introducer, Teleflex).

In the third step, the same experimental cardiopulmonary bypass circuit model was used to investigate the flow capacity of interconnected device components that could form a practically useful endoshunt system; the flow capacity of these interconnected systems was again compared with the observed volume flow rates in the 9 Fr PIS used as a reference standard.

### Experimental step 1

**Figure 1:**
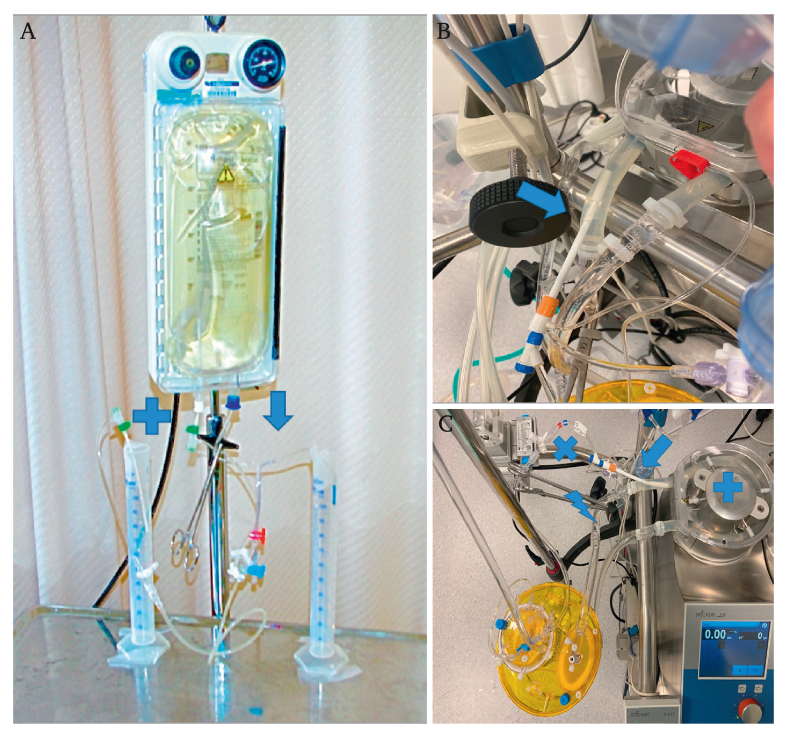
(A) The experimental set up in step 1: tested endoshunt system (+); the internal control (9 Fr Pruit–Inahara shunt) (arrow) (B) A 14 Fr introducer inserted through the silicone tubing (arrow) of the heart lung machine as in experimental steps 2 and 3. (An 8 Fr introducer inserted as the tested component.) (C) The experimental set up in steps 2 and 3, with roller pump (+), silicone tubing, clamp (flash), introducer (arrow) and tested endoshunt system (X)

The volume flow rate capacity of different short and long range ES configurations was investigated in an experimental set up in which the ES volume flow rate was compared with a standard 9 Fr PIS. An intravenous (IV) bag with saline (0.9% sodium chloride) was used as a fluid reservoir. The PIS was connected to the bag through its outlet port, and a surgical clamp was used to interrupt and activate PIS flow. The different investigated ES configurations (Fig. 1A) were inserted through the injection port of the same IV bag using Seldinger technique. The IV bag was pressurised to 90 mmHg to mimic a physiological mean arterial pressure, the valve of the ES and the surgical clamp of the PIS were thereafter opened simultaneously, and the different ES volume flow capacities were sequentially tested for each ES configuration against the PIS shunt capacity (the gold standard). Flow through the two simultaneously tested shunts (i.e., PIS and one of the ES configurations) continued for 30 seconds and the delivered fluid volumes were collected into separate measuring cylinders, enabling calculation of volume flow per minute.

### Experimental step 2

A heart lung machine with roller pumps (Stöckert S5) equipped with the Medex LogiCAl pressure monitoring system (Smith Medical, Plymouth, MN, USA) was used to achieve flow and measure continuous pressure. The disposable circuit consisted of an Inspire hard shell venous reservoir with tubing (LivaNova) primed with Ringer acetate. A hard roller pump occlusion (>500 mmHg) achieved with a dynamic setting was used to ensure a positive flow. A 14 Fr introducer was inserted through the silicone tubing after the roller pump and the pressure monitor. A clamp was applied to the tubing after the introducer, diverting the flow via the 14 Fr introducer and through the tested component as described in (Fig. 1B and C). The flow rate was increased in steps of 40–50 mL/min while monitoring the driving pressure after the roller pump, enabling the creation of pressure–flow curves for the individually tested components.

### Experimental step 3

Based on the results from the two earlier steps, the most favourable endoshunt components in terms of observed flow capacity that could also form a practically useful endoshunt system were selected for a similar test procedure as described in step 2. Pressure–flow curves for the fully integrated endoshunt systems were subsequently created and compared with the corresponding 9 Fr PIS pressure–flow curve. For further comparison and to again fully evaluate how it affected flow capacity, some previously dismissed endoshunt combinations (e.g., a standard 6 Fr introducer and conventional high pressure tubing) were also added in the last experimental step. In total, seven individual inflow and outflow potential ES components were investigated with inflow and outflow diameters ranging from 6 to 15 Fr.

## Results

### Experimental step 1

**Figure 2:**
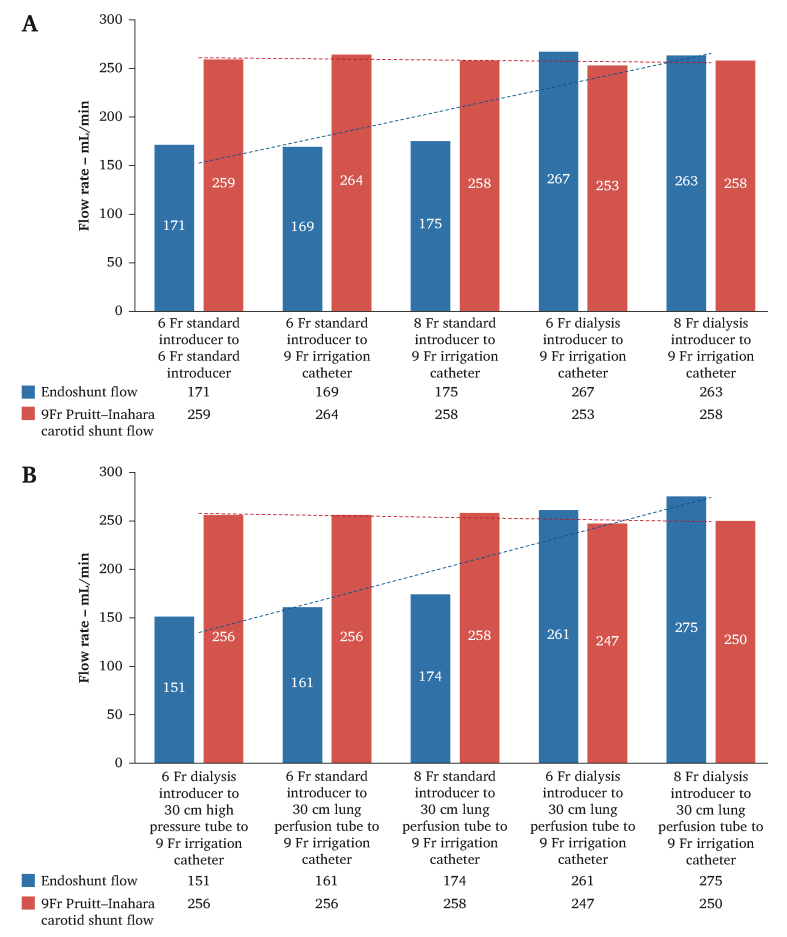
(A) Flow rate in different short distance endoshunt arrangements compared with the corresponding flow rate in a 9 Fr Pruitt–Inahara carotid shunt (internal control). (B) Flow rate in different long distance endoshunt arrangements compared with the corresponding flow rate in a 9 Fr Pruitt–Inahara carotid shunt (internal control)

Fig. 2A,B displays the observed flow rates in the different ES systems compared with the corresponding PIS flow rates. ES systems based on both 6 and 8 Fr standard donor introducers led to a substantially lower volume flow rate than the corresponding PIS volume flow rate, whereas ES systems based on dedicated 6 or 8 Fr dialysis access introducers (without restrictions regarding diameter in the flush port) (Prelude Short Sheet, Merit Medical) matched the PIS flow rates. The introduction of a 30 cm long ¼′′ perfusion tubing between the donor introducer and the recipient 9 Fr irrigation catheter did not affect the volume flow rate for any of the tested ES configurations. The difference between the best performing ES system and the worst was 98 mL/min for short distance ES systems and 124 mL/min for long distance ES systems.

### Experimental step 2

**Figure 3:**
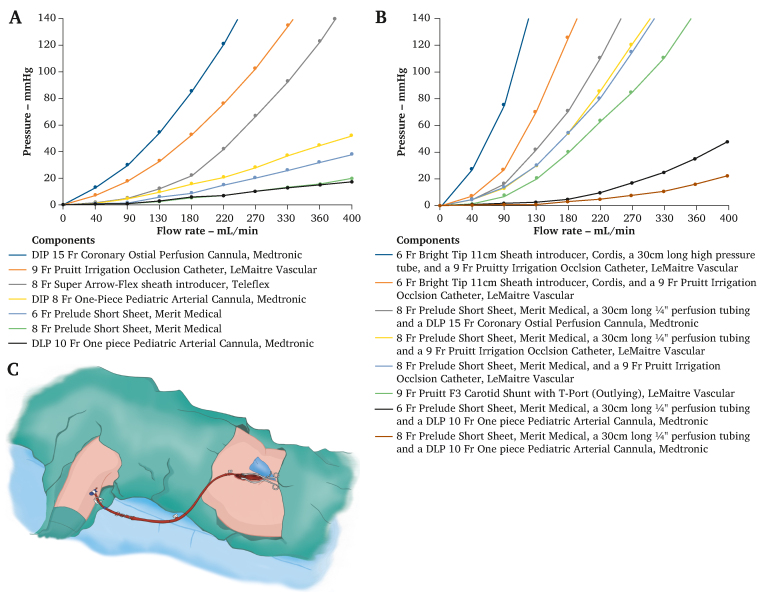
(A) Pressure–flow charts for the individually tested components in experimental step 2. The component with the highest volume flow is the DLP 10 Fr One Piece Paediatric Arterial Cannula (Medtronic) (black). (B) Pressure–flow charts for the tested endoshunt systems in experimental step 3. (C) Schematic illustration of the three component interconnected endoshunt system that had the highest flow capacity in experimental step 3: 8 Fr Prelude Short Sheet (Merit Medical) + 30 cm long ¼′′ perfusion tubing + a DLP 10 Fr One Piece Paediatric Arterial Cannula (Medtronic).

Pressure–flow charts for the individual components tested are presented in Fig. 3A.

### Experimental step 3

Pressure–flow charts for the endoshunt combinations are presented in Fig. 3B.

## Discussion

In this experimental study of the flow capacity of different potential endoshunt components and fully interconnected endoshunt systems, all three experiments indicated that flow optimised endoshunting techniques can match conventional shunts such as the 9 Fr PIS. By applying the principles from the Hagen–Poiseuille equation, assuming laminar flow (*Q* = d*V*/d*t* = π*r*^4^Δ*p*/(8 μL), where Q is the volumetric flowrate, *r* is the radius of the pipe, *L* is the length of the pipe, μ is the dynamic viscosity of the fluid, and Δp is the pressure gradient over the pipe), it can be concluded that the inner radius or diameter will be the most important determinant of flow rate within a pipe system. In the three stage experimental workflow, it was confirmed once again that the most limiting factor to the flow rate within an endoshunt system is the inner diameter of the components used whereas the length of the used components is less important.^6^ For example, by using an 8 Fr Prelude Short Sheet introducer (where the hub of the flush arm is without any diameter restriction) instead of an ordinary 8 Fr introducer a more than doubling of the flow rate was observed within an experimental model mimicking physiological conditions. Another example of this principle was that adding 30 cm of ¼′′ perfusion tubing did not affect the flow rate to any extent.

There have been few studies investigating the required flow rates at a certain arterial driving pressure within a passive shunt system for it to work adequately when applied in a clinical situation. An approximation of appropriate blood flow in different arterial segments was described in an article by Vikatmaa et al. from 2018.^10^ As an example, they suggested a normal renal artery blood flow ranged from between 200 and 400 mL/min and in a common femoral artery to be in between 300 and 1000 mL/min.

Different donor arteries and target organs may require different shunt set ups, due to both vessel diameters and requirements of blood flow. The pressure–flow charts for different endoshunt combinations provided in this article may help tailoring endoshunt systems to different requirements in future applications of the technique.

The experiments also show the importance of the driving pressure in the circuit when using shunt systems, and that this needs to be monitored closely to achieve adequate blood flow output to the target organ. An experimental cardiopulmonary bypass circuit was used to mimic the mean arterial pressure. When considering a realistic mean arterial pressure during an operation, and that many target organs will probably have an opening pressure of at least 20–30 mmHg,^11^^,^^12^ it is unlikely that it will be possible to achieve a driving pressure of more than 50–60 mmHg over the shunt system under physiologically realistic conditions during surgery, and this is an important issue to consider when interpreting the provided pressure–flow charts for the different shunt components and interconnected ES systems explored in this study (Fig. 3A and B). For instance, considering a theoretical scenario involving the shunting of a kidney, if the anaesthetised patient has a mean arterial pressure of 80 mmHg, and assuming an opening pressure for the kidney at 25 mmHg, the renal perfusion pressure that would need to be achieved is 80–25 = 55 mmHg. If the blood flow aimed for is 200–400 mL/min^10^ and the observed experimental values from Fig. 3B are used, it is evident that the Pruitt–Inahara 9F shunt (green) just meets the pressure and flow rate criteria, the (brown) endoshunt system meets them with ease, whereas the (dark blue) shunt system does not meet the criteria. If, for various reasons, it proves challenging to be able to maintain a mean arterial pressure of 80 mmHg throughout the procedure, the selection of shunt system becomes even more important. From Fig. 3B, it can be concluded that the endoshunt system with the highest volume flow is described by the brown curve and is composed of an introducer, 8 Fr Prelude Short Sheet (Merit Medical) 30 cm long ¼′′ perfusion tubing, and a DLP 10 Fr One Piece Paediatric Arterial Cannula (Medtronic). Furthermore, especially when applying longer distance ES shunting techniques in situations where the mean arterial pressure for some reason is lower than optimal, the administration of heparin (if not contraindicated in the specific surgical context) is strongly advised to diminish the risk of shunt thrombosis.

Obvious limitations to this study are that all experiments were undertaken under *in vitro* conditions using Ringer acetate and 0.9% saline solution as the experimental liquid, and that static rather than pulsatile pressure was applied in the experimental circuit. The results would probably have been slightly different if using blood (which has higher viscosity) as the study liquid and within a different experimental set up that generates an oscillating pressure gradient. However, it is believed that these factors would not have a crucial impact on the main findings. Further confirmation of the results reported here within *in vivo* models under physiological conditions is however clearly warranted.

In conclusion, endoshunting techniques can match PIS volume flow rates both over short and long distances when evaluated in a bench test flow circuit with saline as the perfusion medium. The achieved ES flow rates are however highly dependent on the components used in the ES system. The combination of an 8 Fr Prelude Short Sheet introducer (Merit Medical), 30 cm long ¼′′ perfusion tubing (Sorin Group), and a Distal Limb Perfusion 10 Fr One Piece Paediatric Arterial Cannula (Medtronic) had the highest overall flow capacity and would probably be able to meet the metabolic demands of most end organs and tissue beds.

## Conflict of interest

None.

## Funding

This study was supported by grants from the Swedish state under the agreement between the Swedish government and the county councils, the ALF agreement (ALFGBG-785741 and ALFGBG-822921) and the Swedish Heart–Lung Foundation (20190194 and 20200258).
