# Supplementary material for: Optimisation of Volume Flow Rates when Using Endovascular Shunting Techniques: An Experimental Study in Different Bench Flow Circuits
Source: EJVES Vasc Forum. 2022 Nov 19;58:5–10. doi: 10.1016/j.ejvsvf.2022.11.002 (PMC9772544; doi:10.1016/j.ejvsvf.2022.11.002)
Supplement: Multimedia component 1 [file mmc1.docx]

**Supplement**

**The tested individual components presented in figure 3a:**

Dark blue - DLP^®^ 15 Fr Coronary Ostial Perfusion Cannula, Medtronic

Orange - 9 Fr Pruitt^®^ Irrigation Occlusion Catheter, LeMaitre Vascular

Gray - 8 Fr Super Arrow-Flex^®^ sheath introducer, Teleflex

Yellow - DLP^®^ 8 Fr One-Piece Paediatric Arterial Cannula, Medtronic

Light blue - 6 Fr Prelude® Short Sheet, Merit Medical

Green - 8 Fr Prelude® Short Sheet, Merit Medical

Black - DLP^®^ 10 Fr One-Piece Paediatric Arterial Cannula, Medtronic

**The tested endoshunt combinations presented in figure 3b:**

Dark blue - 6 Fr Bright Tip^®^ 11cm Sheath introducer, Cordis, a 30 high pressure tube, and a 9 Fr Pruitt^®^ Irrigation Occlusion Catheter, LeMaitre Vascular

Orange - 6 Fr Bright Tip^®^ 11cm Sheath introducer, Cordis, and a 9 Fr Pruitt^®^ Irrigation Occlusion Catheter, LeMaitre Vascular

Gray - 8 Fr Prelude® Short Sheet, Merit Medical, a 30cm long ¼” perfusion tubing and a DLP^®^ 15 Fr Coronary Ostial Perfusion Cannula, Medtronic

Yellow - 8 Fr Prelude® Short Sheet, Merit Medical, a 30cm long ¼” perfusion tubing and a 9 Fr Pruitt^®^ Irrigation Occlusion Catheter, LeMaitre Vascular

Light blue - 8 Fr Prelude® Short Sheet, Merit Medical, and a 9 Fr Pruitt^®^ Irrigation Occlusion Catheter, LeMaitre Vascular

Green - 9 Fr Pruitt F3^®^ Carotid Shunt with T-Port (Outlying), LeMaitre Vascular

Black - 6 Fr Prelude® Short Sheet, Merit Medical, a 30cm long ¼” perfusion tubing and a DLP^®^ 10 Fr One-Piece Paediatric Arterial Cannula, Medtronic

Brown - 8 Fr Prelude® Short Sheet, Merit Medical, a 30cm long ¼” perfusion tubing and a DLP^®^ 10 Fr One-Piece Paediatric Arterial Cannula, Medtronic
